# Supplementary material for: The Etiology, Antibiotic Therapy and Outcomes of Bacteremic Skin and Soft-Tissue Infections in Onco-Hematological Patients
Source: Antibiotics (Basel). 2023 Dec 13;12(12):1722. doi: 10.3390/antibiotics12121722 (PMC10740481; doi:10.3390/antibiotics12121722)
Supplement: Supplementary file 1 [file antibiotics-12-01722-s001.zip › antibiotics-2724175-supplementary.pdf]

Supplementary Table 1. Clinical features, etiology, antibiotic therapy and outcomes of patients with necrotizing fasciitis.

	**Gender/ Age (years)**	**Underlying disease**	**Risk factors**	**Organism**	**Localization**	**Septic shock**	**Radiological imaging**	**Empirical antibiotic therapy**	**Surgery**	**Outcome**
1	Female/66	Acute leukemia	Steroids	*E. coli*	Limb	Yes	CT	Amoxicillin-clavulanate (inadequate)	No	Death
2	Female/49	Allogeneic HSCT	Steroids	MDR-*P. aeruginosa*	Head and neck	No	CT	Amikacin + colistin	No	ICU admission Death
3	Male/56	Melanoma (Foot)		*S. agalactiae*	Limb	No	CT	Amoxicillin-clavulanate + daptomycin	Yes	Alive
4	Female/57	Acute leukemia	Steroids	*K. pneumoniae*	Limb	No		Carbapenem	No	Death
5	Male/46	Acute leukemia	Neutropenia Steroids Diabetes mellitus	*P. aeruginosa* and *S. mitis*	Limb	Yes	CT /MR	Amoxicillin-clavulanate + daptomycin	Yes	ICU admission Alive
6	Male/75	Prostate cancer	Steroids Diabetes mellitus	*E. coli* and *Bacteroides uniformis*	Limb	No	CT	Carbapenem	No	ICU admission Alive

MDR: multidrug-resistant; CT: computed tomography; MR: magnetic resonance; ICU: intensive care unit.
